# Resolution of Fetal Arrhythmia Following Maternal Discontinuation of Doxylamine (Unisom): A Case Report and Review

**DOI:** 10.7759/cureus.99398

**Published:** 2025-12-16

**Authors:** Olivia L Ponton, Ravi Ashwath, Govinda Paudel

**Affiliations:** 1 Pediatric Cardiology, CHRISTUS Children's Hospital, San Antonio, USA; 2 Pediatric Cardiology, CHRISTUS Children's Hospital, Baylor College of Medicine, San Antonio, USA

**Keywords:** antihistamines, doxylamine, fetal arrhythmia, pregnancy, premature ventricular contraction (pvc)

## Abstract

We report a case of a 36-year-old pregnant woman whose fetus exhibited frequent premature ventricular contractions (PVCs) and occasional probable premature atrial contractions (PACs) at 23 weeks' gestation. The patient was taking Unisom for nausea. After discontinuing the medication, the fetal arrhythmias resolved within a week. Postnatal evaluation showed a normal ECG, a normal QT interval, and no recurrence of arrhythmias. This case highlights the importance of thoroughly reviewing maternal medications when assessing fetal arrhythmia and suggests a possible proarrhythmic effect of doxylamine during pregnancy.

## Introduction

Fetal arrhythmias account for a substantial proportion (10-20%) of referrals to fetal cardiology specialists [1]. Most fetal arrhythmias are benign, commonly presenting as isolated premature atrial contractions (PACs) that resolve spontaneously [1,2]. However, persistent, complex, or ventricular arrhythmias warrant further evaluation to rule out underlying structural, metabolic, infectious, or functional causes. Maternal factors-including systemic illness, electrolyte disturbances, or medication use-can also influence fetal cardiac rhythm [3]. Therefore, a thorough review of maternal history and current medications is an essential component of fetal arrhythmia assessment. 

Antihistamines are sometimes used during pregnancy, owing to their wide availability and established safety record [4]. Doxylamine, a first-generation ethanolamine-class H₁-antihistamine, is commonly taken either as an over-the-counter sleep aid (Unisom) or in combination with pyridoxine to treat nausea and vomiting of pregnancy [4]. Although doxylamine has been considered safe for routine use and carries a low risk of teratogenicity [4], its ability to cross the placenta raises the possibility of direct pharmacologic effects on the fetal cardiovascular system.

This case report describes the normalization of a fetal arrhythmia after Unisom was discontinued in a 36-year-old pregnant woman. The clear temporal association between maternal doxylamine use and the observation of frequent premature ventricular contractions (PVCs) raises the possibility of a medication-related effect. This case adds to the limited evidence on drug-associated fetal arrhythmias and highlights the need to assess maternal medication exposure when evaluating abnormal fetal cardiac rhythms.

## Case presentation

**Figure 1:**
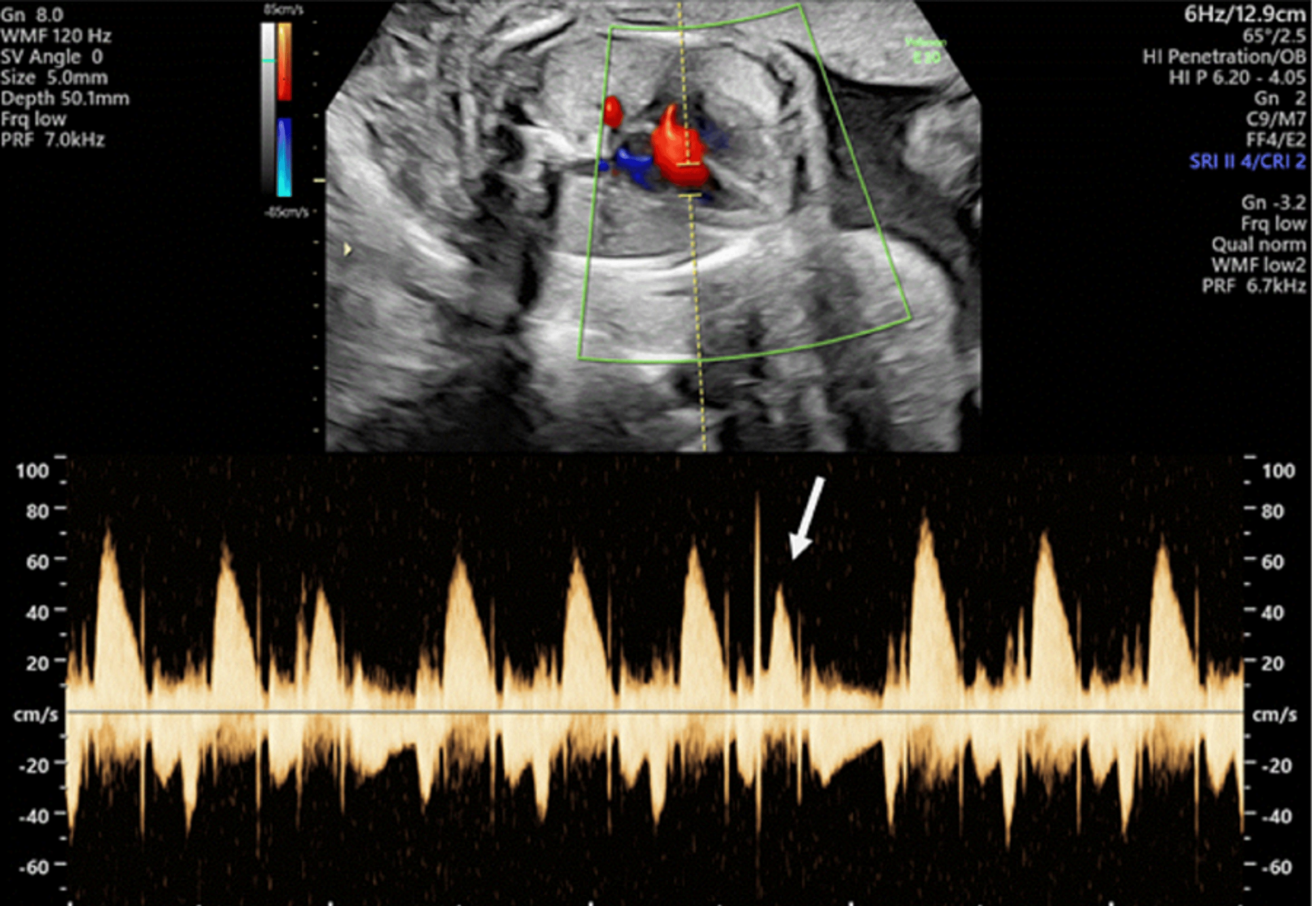
Simultaneous pulsed-wave Doppler of the mitral inflow and left ventricular outflow tract in the fetus, demonstrating a premature beat The premature beat (white arrow) is most consistent with a premature ventricular contraction when correlated with the M-mode findings in Figure 1. A compensatory pause is observed following the premature beat.

**Figure 2:**
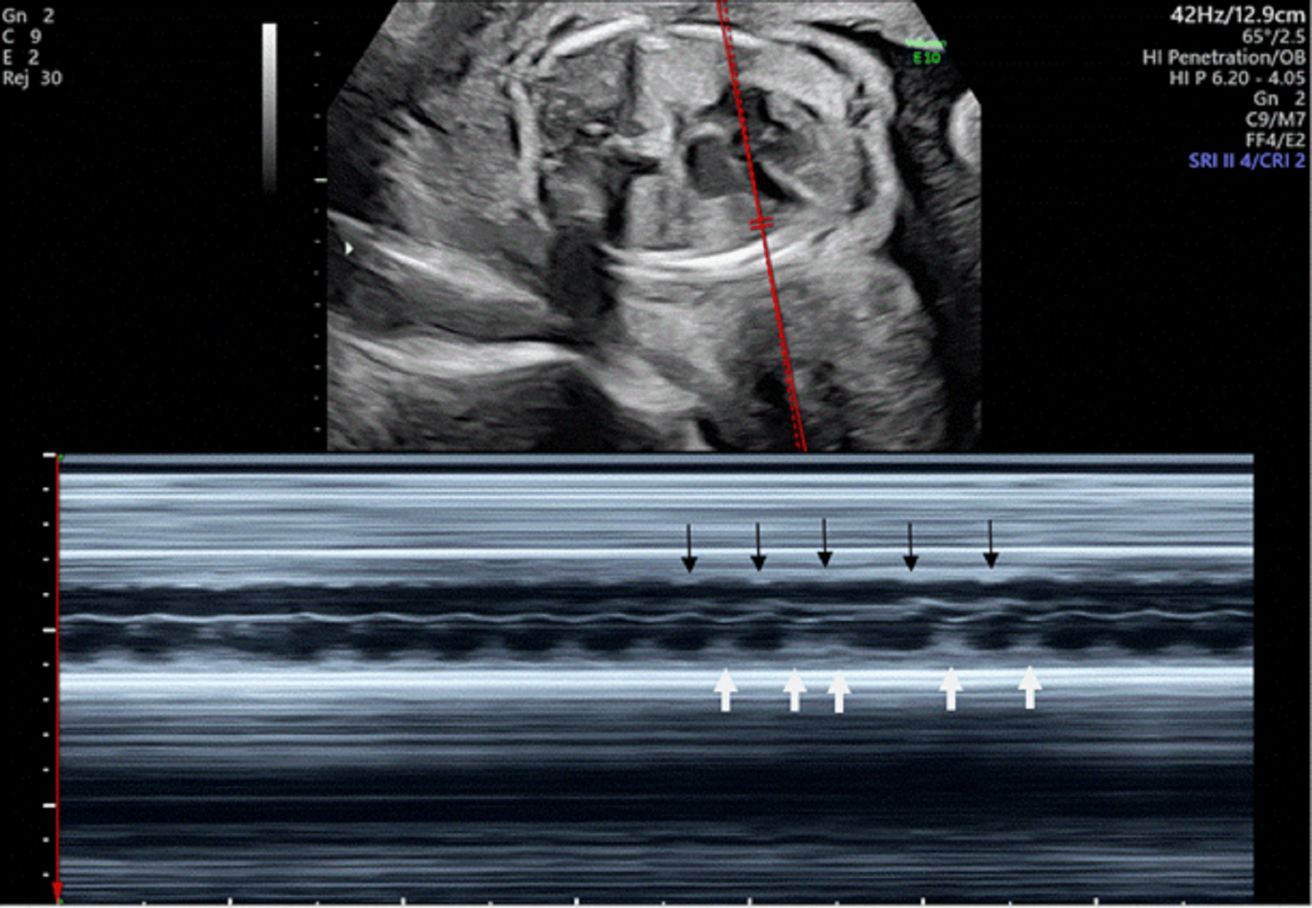
M-mode echocardiographic tracing of the fetal atrium and ventricle demonstrating a premature ventricular contraction Atrial contraction occurs independently of the ectopic ventricular beat. The black arrow denotes an atrial event, and the white arrow denotes a ventricular event. A compensatory pause follows the premature ventricular contraction.

**Figure 3:**
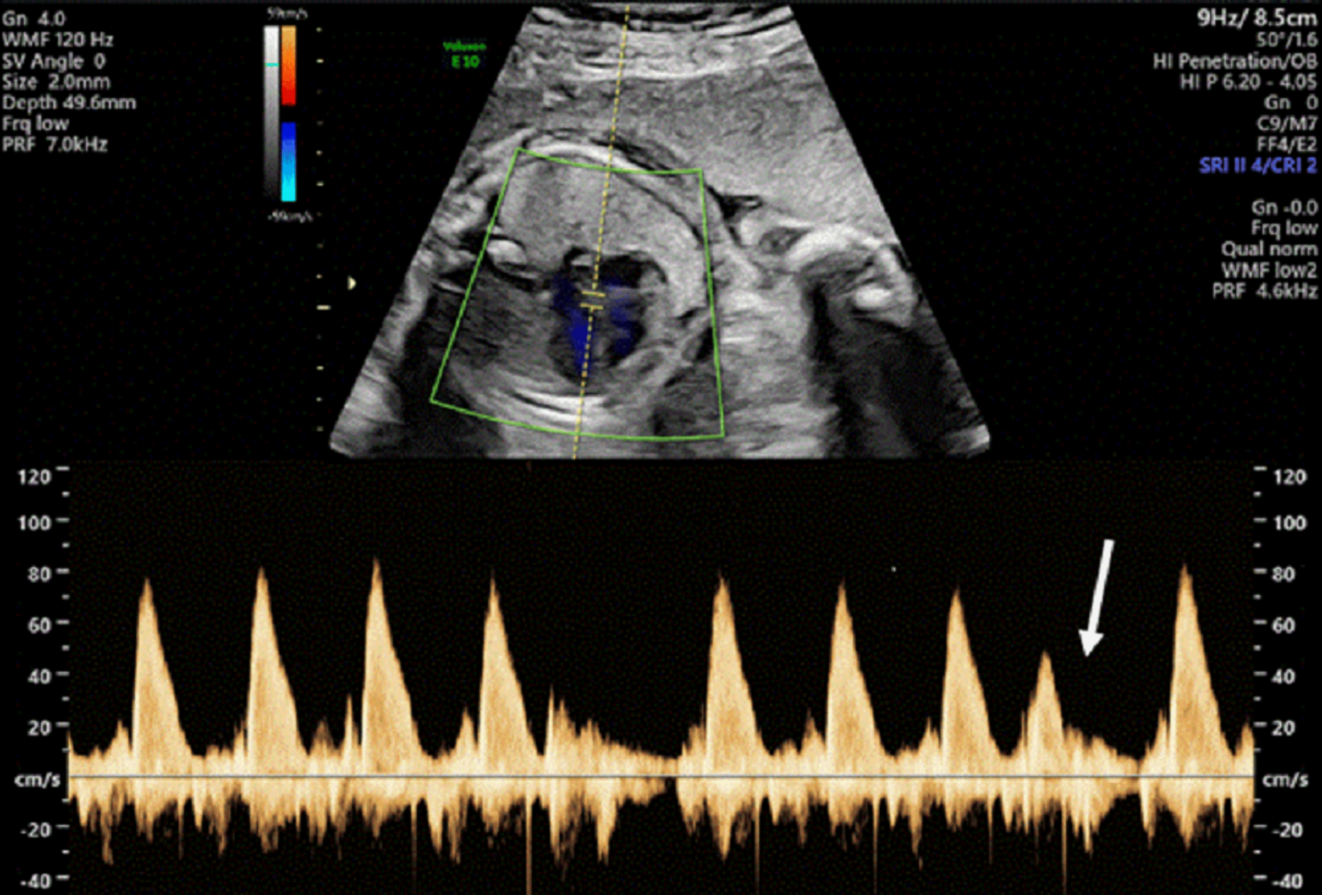
Pulsed-wave Doppler of the fetal left ventricular outflow tract showing a probable premature atrial contraction The premature beat (white arrow) is most consistent with a premature atrial contraction, suggested by the absence of a compensatory pause. However, this observation is not definitive, as the rhythm was intermittent and could not be captured on M-mode, which would have provided definitive confirmation.

A 36-year-old woman, gravida 2 para 1, was referred for maternal-fetal medicine evaluation at 20 weeks’ gestation due to advanced maternal age. Routine fetal assessment detected an irregular cardiac rhythm, prompting referral to fetal cardiology. Fetal echocardiography at 23-week gestation revealed frequent PVCs, shown in Figures 1-2, along with occasional probable premature atrial contractions (PACs), shown in Figure 3.

Cardiac structure and function were otherwise normal. The patient's history was notable for the absence of significant caffeine intake, autoimmune disorders, infection-related symptoms, and any family history of arrhythmias. Complete blood count, urine drug screening, thyroid function test, non-invasive prenatal screening for genetic and chromosomal abnormalities (Panorama test), expanded pan-ethnic carrier screening for genetic conditions, urinalysis, and urine culture and other routine prenatal labs were unremarkable. Her only medications were prenatal vitamins and Unisom for nausea, taken intermittently at therapeutic doses on most days for more than three weeks prior to this visit.

**Figure 4:**
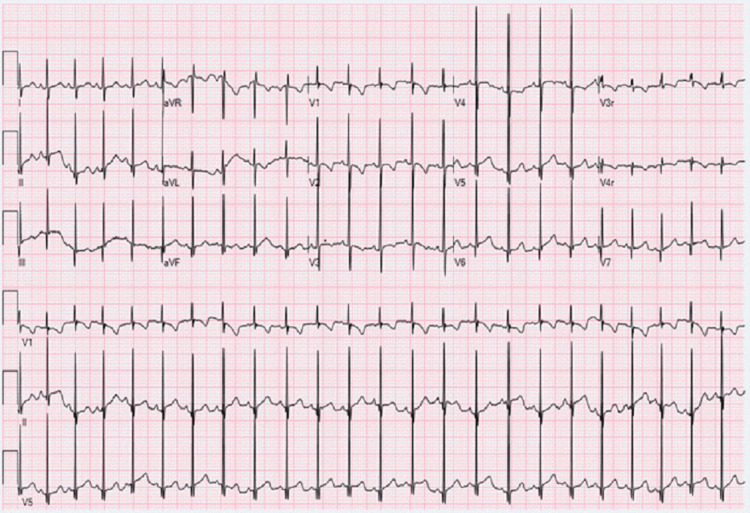
Standard electrocardiogram of the 2-month-old infant demonstrating normal sinus rhythm, normal QTc interval, and no evidence of premature beat The EKG demonstrates the ventricular and atrial rates being 142 bpm. PR interval: 98 ms; QRS duration: 54 ms; QT interval: 260 ms; QTc: 399 ms. PRT axis: 40°, 69°, and 58°, X-axis 25mm/s, Y-axis: 10mm/mv.

Although evidence regarding fetal susceptibility is limited, prior reports indicate a possible association between first-generation antihistamines and arrhythmias in children [5]. Given this concern, the patient was advised to discontinue Unisom. At the one-week follow-up, fetal echocardiography demonstrated complete resolution of the arrhythmia, with a persistently normal rhythm on subsequent assessments. Postnatal evaluation, including ECG as illustrated in Figure 4, confirmed normal QTc interval, normal cardiac rhythm, and structure.

## Discussion

Fetal rhythm abnormalities, which include fetal heart rates that are irregular, too fast, or too slow, occur in up to 2% of pregnancies and account for 10-20% of the referrals to fetal cardiologists [1]. Irregular heart rhythms secondary to ectopic beats are the most common abnormal rhythms seen in clinical practice. Ectopic beats are usually atrial in origin, though occasionally they are ventricular in origin [6]. The prevalence of fetal PACs compared with PVCs is roughly 10:1 [7]. Most fetal arrhythmias are benign and require only monitoring. However, persistent or complex rhythms may signal underlying structural, functional, or metabolic abnormalities and can occasionally progress to more significant disturbances. PVCs are generally considered more concerning than premature atrial contractions. The evaluation of fetal arrhythmia requires a systematic approach, including detailed maternal history, fetal echocardiography, and assessment for secondary causes such as maternal medications. This case underscores the critical importance of reviewing maternal medications when evaluating fetal arrhythmia. Although most premature beats in the fetus are benign and resolve spontaneously, secondary causes, including maternal drug exposure, should be considered.

Doxylamine, the active ingredient in Unisom, is an ethanolamine-class first-generation H1-antihistamine. Although doxylamine-pyridoxine is the only FDA-approved therapy for nausea and vomiting in pregnancy and is generally considered safe at standard doses [4], like other first-generation antihistamines, doxylamine is known to cross the placenta. Its lipophilic properties and low molecular weight facilitate placental transfer, raising the possibility of pharmacologic effects on the fetus. Most existing research on first-generation H₁-antihistamines has concentrated on their cardiovascular effects in pediatric populations, with limited data regarding fetal exposure. A notable case-crossover study in a pediatric population conducted in Korea found a 20% increased risk of cardiovascular events, including arrhythmias, in children exposed to first-generation H1-antihistamines [5]. Another study reported an association between doxylamine use in pregnancy and an increased risk of hypoplastic left heart syndrome (HLHS) in offspring, with an adjusted Odds Ratio of 1.73 (95% CI: 1.09-2.76) [8]. Case reports in adults have documented long QT intervals, arrhythmias, and cardiomyopathy with doxylamine overdose, although such reactions are exceptionally uncommon at standard doses [9]. First-generation antihistamines may provoke arrhythmias by blocking cardiac potassium channels, resulting in QT prolongation and a higher risk of ectopic activity. Comparable electrophysiologic effects have been reported with other drugs in the same class. For example, Paudel et al. reported a case of pyrilamine-induced QT interval prolongation in an adolescent following drug overdose, further supporting the potential proarrhythmic effects of this class of medications [10]. Additional reports have described QT prolongation and torsade de pointes associated with the use of other antihistamines as well [11,12].

The absence of systemic illness, normal fetal echocardiographic findings without structural or functional abnormalities, and lack of other identifiable etiologies for fetal arrhythmia, along with normalization of fetal rhythm within one week after discontinuation of doxylamine in this case, suggest a possible transient in-utero medication effect.

## Conclusions

This case highlights the need to carefully assess maternal medications when evaluating fetal arrhythmias. The fetal rhythm normalized within a week of discontinuing doxylamine, and postnatal ECG showed a normal QTc without recurrence of arrhythmia, suggesting a possible transient in-utero medication effect. Although doxylamine is generally considered safe in pregnancy, this report raises awareness of its potential proarrhythmic properties. Current guidelines emphasize identifying secondary causes of fetal arrhythmia, including maternal drug exposure. Clinicians should therefore remain vigilant when a temporal association is noted. While some fetal arrhythmias resolve spontaneously, the prompt improvement after stopping doxylamine supports a possible drug-related mechanism that merits further study.
